# Natural Products Targeting Acetylation in Bladder Cancer: Mechanistic Basis, Therapeutic Potential, and Future Perspectives

**DOI:** 10.3390/cimb48050489

**Published:** 2026-05-08

**Authors:** Wei Li, Da Liu, Qinzhamusu Yin, Yiwen Geng, Yang Liu, Yong Wang

**Affiliations:** 1 College of Pharmacy, Changchun University of Chinese Medicine, Changchun 130117, China; lw17638310029@163.com (W.L.); liuda@ccucm.edu.cn (D.L.); 13844846135@163.com (Y.G.); 2 Public Experimental Center, Changchun University of Chinese Medicine, Changchun 130117, China; 3 School of Clinical Medicine, Changchun University of Chinese Medicine, Changchun 130117, China; qinzhamusuyin@ccucm.edu.cn; 4 College of Traditional Chinese Medicine, Changchun University of Chinese Medicine, Changchun 130117, China

**Keywords:** natural product, acetylation, bladder cancer, epigenetic modification

## Abstract

Bladder cancer remains a major clinical challenge because of its high recurrence rate, marked molecular heterogeneity, frequent progression, and limited durability of current therapeutic strategies. Increasing evidence indicates that acetylation, as a reversible and druggable epigenetic modification, plays a central role in bladder cancer biology by linking chromatin remodeling to transcriptional regulation, DNA damage repair, metabolic adaptation, and immune modulation. Both histone and non-histone acetylation are frequently dysregulated in bladder cancer, and these alterations contribute to multiple malignant phenotypes, including sustained proliferation, defective cell-cycle control, apoptosis evasion, epithelial–mesenchymal transition, metastatic progression, and therapeutic resistance. In this review, we summarize the mechanistic basis of acetylation imbalance in bladder cancer, with particular emphasis on the roles of histone acetyltransferases, histone deacetylases, sirtuins, and acetylation-associated metabolic regulators. We further discuss the emerging evidence that natural products can modulate acetylation-related pathways in bladder cancer, mainly through targeting HDAC-dependent histone deacetylation and SIRT1-associated non-histone deacetylation. Representative compounds, including sulforaphane, erucin, puerarin, capsaicin, curcumin, trichostatin A, trichostatin C, and pinocembrin, highlight the potential of natural products to suppress tumor growth, promote apoptosis, impair migration, and enhance antitumor immunity through acetylation-related mechanisms. Beyond summarizing individual agents, the evidence was evaluated based on the integration of acetylation-related target engagement, acetylation remodeling, and bladder cancer-relevant phenotypic outcomes. The current evidence is heterogeneous. SFN/ECN, capsaicin, and pinocembrin offer the most convincing bladder cancer-specific support, whereas several other compounds remain limited by context-dependent effects, indirect pathway inference, or incomplete validation of the proposed acetylation mechanisms. These findings support an evidence-oriented translational framework that prioritizes natural products according to mechanistic robustness, bladder cancer specificity, and combination potential. Overall, acetylation-targeting natural products represent a promising but still evolving therapeutic strategy for bladder cancer, warranting further subtype-specific, mechanistically rigorous, and translationally oriented investigation.

## 1. Introduction

Bladder cancer (BCa) is one of the most common malignancies of the urinary system worldwide and is characterized by high incidence, substantial mortality, frequent recurrence, and marked molecular heterogeneity [1]. Based on clinicopathological features and distinct evolutionary trajectories, BCa is broadly classified into non-muscle-invasive bladder cancer (NMIBC) and muscle-invasive bladder cancer (MIBC) [2,3]. Although the majority of patients initially present with NMIBC, which is generally associated with a relatively lower grade of malignancy, its high recurrence rate, potential for progression to invasive disease, and the limited durability of current therapeutic options in advanced settings continue to compromise long-term disease control [4,5]. These challenges underscore the urgent need to develop mechanism-based therapeutic strategies that go beyond conventional cytotoxic approaches.

Accumulating evidence has established that epigenetic dysregulation plays a pivotal role in cancer initiation, progression, and therapeutic response [6]. Epigenetic regulation mainly encompasses DNA methylation, histone and non-histone acetylation, as well as non-coding RNA-mediated post-transcriptional regulation [7]. Although these modifications do not alter the DNA sequence itself, they can profoundly influence tumor cell behavior by reshaping chromatin architecture, transcriptional programs, and signaling networks [8,9]. Among these epigenetic mechanisms, acetylation warrants particular attention because it lies at the intersection of chromatin accessibility and signal-responsive protein regulation. In addition to remodeling transcriptional output, acetylation also affects the activity, stability, and subcellular localization of a wide range of proteins [10]. Notably, BCa is considered one of the solid tumors most frequently affected by alterations in chromatin-regulatory genes, including EP300/CREBBP, KDM6A, and ARID1A [11,12]. In particular, aberrations in EP300/CREBBP, which impair histone acetyltransferase (HAT) function, have been associated with aggressive biological behavior, suggesting that dysregulated acetylation is not merely a bystander event but rather a functionally relevant layer of bladder cancer biology [13].

Natural products are structurally diverse bioactive compounds derived from microorganisms, plants, animals, and marine organisms, and they have long served as a valuable resource for disease treatment and drug discovery [14]. In recent years, a growing body of evidence has demonstrated that numerous natural products and their derivatives possess potent anticancer activities, with some having already been successfully translated into clinical therapeutics, such as paclitaxel [15]. With the continued advancement of epigenetic therapy, acetylation has emerged as a particularly attractive target because of its reversibility and druggability. A variety of natural compounds have been reported to modulate HATs, histone deacetylases (HDACs), sirtuin family deacetylases, or the acetylation status of critical substrate proteins through multitarget mechanisms, thereby exerting antitumor effects [16]. This is especially relevant in cancer, where acetylation is intricately linked not only to chromatin opening and transcriptional reprogramming, but also to DNA damage repair, therapeutic resistance, autophagy, and immune evasion [17,18,19]. Therefore, systematically summarizing the current progress in natural products targeting acetylation in BCa may deepen our understanding of the epigenetic basis of bladder cancer and provide a conceptual framework for the development of novel therapeutic strategies. In the present review, however, bladder cancer-based studies are treated as the primary line of evidence, whereas observations from other malignancies are used only to provide mechanistic context, indicate biological plausibility, or highlight unresolved questions rather than to serve as equivalent proof in BCa.

The added value of this review lies not only in summarizing acetylation-related mechanisms and natural products, but also in organizing the evidence according to mechanistic rigor and translational relevance. Specifically, this review distinguishes bladder cancer-specific evidence from cross-cancer mechanistic background, evaluates whether each compound establishes a coherent chain from target engagement to acetylation remodeling and antitumor phenotype, and highlights key inconsistencies, limitations, and therapeutic implications in the current literature. This evidence-weighted framework may provide a more informative basis for developing acetylation-targeting natural products for bladder cancer.

## 2. Acetylation Dysregulation as an Epigenetic Mechanism in Cancer

Oncogenes and tumor suppressors are established drivers of tumor initiation and progression, and their dysregulation profoundly influences cancer cell proliferation, survival, and therapeutic response [20]. Notably, the activity of these key molecules and their downstream signaling outputs are frequently modulated by acetylation. For instance, p300/CREBBP-associated factor (PCAF) has been reported to suppress protein kinase B (AKT) phosphorylation by promoting histone H4 acetylation, thereby inducing apoptosis in hepatocellular carcinoma cells [21]. Consistently, HDAC1, a major deacetylase, is overexpressed in prostate cancer, and the bitter melon seed-derived protein MCP30 has been shown to inhibit HDAC1 activity, increase H3 and H4 acetylation, suppress AKT phosphorylation, and ultimately trigger tumor cell apoptosis [22]. Beyond its role in canonical oncogenic signaling, acetylation dysregulation can also influence the tumor immune microenvironment. In bladder cancer, for example, HDAC7 activity has been implicated in restricting cluster of differentiation 8-positive (CD8+) T-cell infiltration and reducing tumor sensitivity to programmed death-ligand 1 (PD-L1)-directed immunotherapy [23]. In addition, the oncogenic protein c-Myc can be acetylated by PCAF at K323 and K417, a modification that promotes its stability and protects it from degradation [24]. Collectively, these findings suggest that acetylation is not only a central component of epigenetic regulation, but also a critical mechanism governing the stability, activity, and functional output of multiple cancer-related proteins.

To provide a clearer overview of the oncogenic significance of acetylation imbalance, Table 1 summarizes representative regulators or acetylation-associated markers, their direct substrates, concise mechanisms, and biological consequences in different cancer settings. This comparison underscores that abnormal acetylation is not merely a molecular alteration, but a functionally important event that reshapes multiple aspects of tumor progression.

Several trends emerge from Table 1. First, acetylation-related regulators repeatedly converge on a limited set of cancer-relevant processes, including proliferative signaling, cell-cycle control, apoptosis, DNA damage repair, metastasis, and therapeutic resistance. Second, regulators within the same broad enzyme class do not necessarily produce equivalent biological outcomes. Acetyltransferase-dependent mechanisms may preserve tumor-suppressive transcriptional programs or, in other contexts, enhance oncogenic signaling depending on the substrate and tumor type. Similarly, HDAC- or sirtuin-associated regulation may promote tumor progression by reinforcing survival, motility, or DNA repair, but may also create therapeutic vulnerabilities when excessive deacetylation suppresses pro-apoptotic or checkpoint pathways. Third, the table highlights a major knowledge gap: many studies report altered expression or activity of acetylation regulators, whereas fewer establish a complete enzyme–substrate–site–phenotype chain. Future work should therefore move beyond global acetylation changes and define the specific acetylation events that are functionally required for individual malignant phenotypes.

### 2.1. Histone Acetylation Imbalance

Histone acetylation is a central determinant of chromatin accessibility and transcriptional competence. By neutralizing the positive charge of lysine residues on histone tails, acetylation weakens histone–DNA interactions and promotes a more permissive chromatin state [38,39]. Given its central role in determining chromatin accessibility, disruption of histone acetylation is frequently implicated in tumorigenesis [40]. In cancer, disruption of this balance contributes to epigenetic reprogramming, frequently resulting in inappropriate silencing of tumor suppressor genes or activation of oncogenic transcriptional programs. Histone acetylation imbalance is therefore not merely a biochemical abnormality, but a recurrent mechanism through which tumor cells remodel gene expression [41,42].

The histone acetylation landscape is dynamically controlled by HATs and HDACs, and its disruption is widely observed across tumor types. Importantly, the consequences of this dysregulation are highly context dependent, reflecting differences in enzyme activity, acetylation-site specificity, and tumor lineage [43]. Genomic analyses have identified recurrent alterations in chromatin-modifying genes, including EP300 and other HAT-related components, which are associated with impaired acetyltransferase activity and insufficient histone acetylation, thereby facilitating the epigenetic silencing of tumor suppressor genes [44,45]. In addition to abnormalities in acetylation-regulating enzymes, disruption of specific histone acetylation marks also contributes to tumor progression. For example, elevated H2AK5ac has been observed in HeLa cells, whereas HAT1 knockdown suppresses tumor cell proliferation [25]. Moreover, reduced expression of H3K4ac and H3K9ac has been reported in oral squamous cell carcinoma and ovarian cancer, where these changes are closely associated with tumor stage and poor prognosis [46,47]. H4K16ac, a key acetylation site on histone H4, exhibits striking context-dependent patterns across different malignancies. In non-small-cell lung cancer (NSCLC), both H4K16ac and its associated acetyltransferase hMOF are highly expressed, and hMOF has been shown to promote S-phase entry through regulation of SKP2, thereby contributing to NSCLC development [26,48]. By contrast, reduced H4K16ac has been documented in renal cell carcinoma, breast cancer, and colorectal cancer [49,50,51].

In addition to defective HAT activity, aberrant HDAC function represents another major driver of histone acetylation imbalance in cancer [52]. Based on sequence homology, catalytic mechanisms, and functional characteristics, the HDAC family is generally classified into four groups: class I (HDAC1, HDAC2, HDAC3, and HDAC8), class II (HDAC4, HDAC5, HDAC6, HDAC7, HDAC9, and HDAC10), class III (the sirtuin family, including SIRT1–SIRT7), and class IV (HDAC11) [53]. Notably, the functions of individual HDACs are highly context-dependent across tumor types. For instance, reduced HDAC2 expression in colon cancer has been associated with upregulation of the H19/MMP14 axis, thereby promoting metastasis and epithelial–mesenchymal transition (EMT) [27]. In contrast, HDAC2 is frequently overexpressed in bladder cancer and is associated with enhanced proliferation, metastasis, and chemoresistance [54,55]. Similarly, HDAC1 has been reported to be upregulated in gastric cancer, and its knockdown markedly suppresses malignant proliferation [28]. In addition, both HDAC8 and HDAC10 can deacetylate AKT1, thereby enhancing its phosphorylation signaling and promoting tumorigenesis [29,30]. Meanwhile, SIRT7 knockdown has been shown to decrease cell viability and induce apoptosis [56]. Taken together, these observations suggest that impaired HAT function and aberrant HDAC activity collectively reshape the histone acetylation landscape and constitute an important epigenetic mechanism driving bladder cancer progression.

### 2.2. Non-Histone Acetylation Imbalance

The regulatory scope of acetylation extends far beyond histones. A broad range of non-histone proteins—including transcription factors, signaling mediators, and cytoskeletal regulators—are also subject to reversible acetylation, which can alter their stability, localization, DNA-binding activity, and interactions with partner proteins [57,58]. Through these effects, non-histone acetylation links epigenetic machinery to intracellular signaling and stress adaptation, thereby expanding the oncogenic consequences of acetylation dysregulation beyond chromatin itself.

#### 2.2.1. Growth- and Survival-Related Signaling

One major consequence of non-histone acetylation imbalance is the aberrant regulation of growth- and survival-related signaling [59]. Acetylation-dependent modulation of key nodes within the PI3K/AKT/mTOR and MAPK pathways can reshape proliferative signaling, stress adaptation, and resistance to cell death, thereby conferring a survival advantage to tumor cells.

The phosphatidylinositol 3-kinase/protein kinase B/mechanistic target of rapamycin (PI3K/AKT/mTOR) and mitogen-activated protein kinase (MAPK) pathways are two of the most important signaling axes controlling cancer cell growth and survival [60]. The former is typically initiated by growth factor-induced activation of receptor tyrosine kinases, which recruit and activate PI3K, promote phosphatidylinositol (3,4,5)-trisphosphate (PIP3) generation, and subsequently enable AKT activation, ultimately regulating mTOR-dependent protein synthesis, cell-cycle progression, and anti-apoptotic signaling [61]. By contrast, the MAPK pathway is generally triggered through receptor-mediated activation of Ras, followed by sequential phosphorylation of rapidly accelerated fibrosarcoma (RAF), MAPK/ERK kinase (MEK), and extracellular signal-regulated kinase (ERK), thereby modulating a broad range of nuclear and cytoplasmic effectors involved in proliferation, differentiation, and stress responses [62]. Because the activity of these pathways depends on the functional state of central signaling proteins, regulatory inputs beyond phosphorylation are also highly relevant. Emerging evidence indicates that acetylation can modify pathway output at multiple levels, adding an additional layer of control to canonical oncogenic signaling.

Consistent with this view, dysregulated non-histone acetylation can promote tumor progression by altering the functional state of signaling proteins within canonical oncogenic pathways such as PI3K/AKT and MAPK.

KAT6A, a member of the MYST family of lysine acetyltransferases, has been shown to catalyze H3K23 acetylation and recruit the H3K23ac reader TRIM24, thereby activating PIK3CA transcription, enhancing PI3K/AKT signaling, and promoting tumorigenesis [31]. In parallel, NAT10, a GCN5-related N-acetyltransferase family member, was reported to acetylate BCL-XL mRNA in multiple myeloma, resulting in activation of the PI3K/AKT pathway and increased proliferation of myeloma cells [32]. In addition to acetyltransferase-driven regulation, metabolism-linked deacetylation mechanisms may also influence this signaling axis. For example, PARP1 inhibition increases intracellular NAD+ levels and activates SIRT1, thereby promoting AKT deacetylation and revealing an inverse correlation between AKT acetylation and phosphorylation [33].

A similar regulatory logic also applies to the MAPK pathway, whose signaling output appears to be modulated, at least in part, by the acetylation status of ERK-associated machinery. Available evidence indicates that HDAC6 deacetylates ERK1/2 and enhances its kinase activity, whereas HDAC6 inhibition leads to increased acetylation of endogenous ERK1/2, suggesting that the acetylation status of ERK1/2 can directly influence MAPK signaling output [63,64]. On this basis, combined inhibition of HDAC6 and ERK1/2 has been proposed as a potential strategy to overcome resistance to epidermal growth factor receptor (EGFR)-, RAF-, or MEK-targeted therapies. Taken together, these findings support the view that acetylation extends far beyond chromatin regulation and can profoundly reprogram tumor cell growth and survival signaling through direct modulation of specific signaling proteins and their associated regulatory networks.

#### 2.2.2. p53- and Cell-Cycle-Related Signaling

Another important consequence of non-histone acetylation imbalance is the dysregulation of p53-dependent stress responses and cell-cycle checkpoint control [65]. In contrast to growth-promoting pathways such as PI3K/AKT/mTOR and MAPK, which primarily support proliferation and survival, the p53 and retinoblastoma protein (RB)/E2F axes function as major restraints on aberrant cell-cycle progression and genomic instability [66,67]. Accordingly, acetylation-dependent disruption of these pathways may not only attenuate tumor-suppressive responses, but also facilitate unchecked proliferation and stress tolerance in cancer cells.

Among non-histone acetylation substrates, p53 is one of the best-characterized examples. Multiple lysine acetyltransferases, including p300/CREB-binding protein (CBP), PCAF, general control non-derepressible 5 (GCN5), and TIP60, acetylate distinct lysine residues within p53, thereby modulating its stability, promoter binding, transcriptional activity, and interactions with regulatory partners [68]. Functionally, p53 acetylation is closely linked to the transcriptional activation of genes involved in cell-cycle arrest, senescence, DNA repair, and apoptosis. Notably, TIP60-dependent acetylation of p53 at K120 has been shown to influence the choice between cell-cycle arrest and apoptosis, highlighting that acetylation can shape not only the magnitude but also the qualitative output of p53 signaling [34].

Accordingly, impaired p53 acetylation has emerged as an important mechanism by which tumor cells attenuate stress responses and escape apoptosis. For example, one study showed that PP2Cδ suppresses p300-mediated p53 acetylation through the ATM/BRCA1 pathway, thereby promoting tumor progression [36]. Another study found that within the Smad1–p53–p300 complex, Smad1 inhibits p300-mediated acetylation of p53 while enhancing its own acetylation, ultimately driving tumor progression and therapeutic resistance. Notably, this phenotype could be reversed by a small-molecule compound that interferes with p300-mediated acetylation of Smad1 at K373 [37]. In contrast, restoration of p53 acetylation appears to exert clear antitumor effects. In an A375 melanoma xenograft model, ginsenoside Rg3 in combination with the potent HDAC3 inhibitor MS-275 was shown to enhance p53 acetylation at K373/K382, leading to suppression of xenograft tumor growth [69]. Similarly, an RNA interference (RNAi)-based nanoparticle targeting HDAC2 was reported to increase p53 acetylation and inhibit the growth of both murine xenografts and human hepatocellular carcinoma [70].

The influence of non-histone acetylation is not limited to p53, but also extends to other core regulators of cell-cycle progression and DNA damage signaling. A representative example is E2F1, whose acetylation enhances its transcriptional activity, protein stability, and promoter selectivity, thereby linking acetylation to both checkpoint regulation and apoptotic commitment [71]. E2F1 can be acetylated by lysine acetyltransferases such as PCAF, p300/CBP, and TIP60 on lysine residues adjacent to its DNA-binding domain, and this modification has been shown to enhance its DNA-binding capacity, transcriptional activity, and protein stability [72].

Further support for this model comes from studies showing that E2F1 acetylation is tightly connected to the DNA damage response and cisplatin sensitivity. For example, TIP60 has been shown to promote E2F1 acetylation and to contribute to the upregulation of excision repair cross-complementation group 1 (ERCC1), a key enzyme involved in cisplatin-induced DNA damage repair [35]. This finding suggests that TIP60 may enhance DNA repair capacity, at least in part, through acetylation-dependent activation of E2F1. Another study, by comparing the distinct roles of PCAF and p300 in maintaining E2F1 acetylation and transcriptional stability, further supported a functional connection between E2F1 acetylation and the DNA damage response [73]. Notably, increased E2F1 acetylation was shown to selectively recruit E2F1 to the p73 promoter, thereby triggering apoptotic signaling [74]. In addition, histone deacetylase inhibitors (HDACis) have been reported to enhance E2F1 acetylation, leading to miR-149-mediated repression of ERCC1 and ultimately increasing cisplatin sensitivity in NSCLC cells [75].

Taken together, these findings indicate that non-histone acetylation imbalance can reprogram tumor cell fate not only by strengthening mitogenic signaling, but also by weakening the acetylation-dependent control of checkpoint and stress-response pathways. Through its effects on DNA repair, apoptotic commitment, and therapeutic responsiveness, acetylation occupies a central position at the intersection of proliferation, genomic maintenance, and adaptation to stress.

## 3. Dysregulation of Acetylation in Bladder Cancer

In bladder cancer, dysregulated acetylation has emerged as a mechanistically relevant layer of tumor biology rather than a secondary epigenetic byproduct. Alterations in both histone and non-histone acetylation affect chromatin accessibility, transcriptional control, and the function of key signaling proteins, thereby shaping multiple malignant traits [76]. As a result, acetylation imbalance contributes to proliferation, apoptosis evasion, phenotypic plasticity, metastatic progression, and treatment resistance [77]. Representative regulators and their downstream consequences in bladder cancer are summarized in Table 2.

Acetylation dysregulation in bladder cancer should not be interpreted as a unidirectional oncogenic or tumor-suppressive event. Its biological consequence depends on the specific writer or eraser involved, the substrate being modified, the lysine residue affected, and the molecular context of the tumor. This distinction is particularly important in bladder cancer, which is characterized by marked intertumoral heterogeneity, recurrent alterations in chromatin-regulatory genes, and clinically relevant differences between NMIBC and MIBC as well as among luminal-like, basal-like, immune-infiltrated, and treatment-resistant states. Therefore, generalized descriptions such as “increased acetylation” or “decreased acetylation” are insufficient unless they are linked to defined enzyme–substrate–site relationships and disease contexts.

Table 2 reveals several disease-specific features of acetylation dysregulation in bladder cancer. First, the current evidence is concentrated around a relatively small group of regulators, including EP300/KAT8, HDAC2/6/7, SIRT1/7, and acetyl-CoA-associated metabolic enzymes such as ACAT1 and ACSL5. This suggests that acetylation dysregulation in bladder cancer may be better understood as a set of discrete functional circuits rather than as a uniform global shift in acetylation levels. Second, these regulators map onto several clinically relevant phenotypes: EP300 and KAT8 are linked to chromatin regulation and tumor progression; HDAC2, HDAC6, and SIRT7 are associated with proliferation, migration, EMT, and chemoresistance; SIRT1 is connected to stress adaptation and autophagy; HDAC7 links acetylation regulation to immune exclusion and reduced immunotherapy sensitivity; and ACAT1/ACSL5 illustrate the interface between metabolism and acetylation-dependent tumor control. Third, the therapeutic implications differ among these nodes. HDAC- and SIRT-centered vulnerabilities currently appear most directly actionable, whereas HAT-associated alterations and acetyl-CoA-linked metabolic circuits require more precise functional validation before they can be translated into treatment strategies. Finally, the table also exposes major knowledge gaps, including limited site-specific acetylation mapping, insufficient subtype-resolved analysis, and relatively few studies using patient-derived, orthotopic, or therapy-resistant bladder cancer models.

### 3.1. Histone Acetylation Imbalance in Bladder Cancer

In bladder cancer, mounting evidence indicates that both HATs and HDACs are aberrantly regulated, collectively driving an imbalanced histone acetylation landscape. This imbalance is commonly characterized by reduced HAT activity together with enhanced HDAC-dependent deacetylation, ultimately favoring transcriptional repression. Genomic analyses have identified recurrent alterations in chromatin-modifying genes, including EP300 and other HAT-related components, which are associated with impaired acetyltransferase activity and insufficient histone acetylation, thereby facilitating the epigenetic silencing of tumor suppressor genes [44]. At the same time, certain acetylation-related pathways may also support tumor progression; for example, pharmacological inhibition of KAT8 by MG149 reduces acetylation of its downstream target YEATS4 and suppresses bladder cancer cell viability [78]. Meanwhile, overexpression or hyperactivation of HDACs further reinforces chromatin condensation and transcriptional repression. Increased HDAC activity has been linked to reduced expression of genes involved in cell-cycle control and apoptosis [84]. Notably, HDAC2 overexpression promotes proliferation, metastasis, and chemoresistance, whereas SIRT7 knockdown decreases cell viability and induces apoptosis [54,85]. Taken together, these observations underscore that the combined effects of impaired HAT function and heightened HDAC activity constitute an important epigenetic driver of bladder cancer progression.

In this context, impaired HAT activity should be interpreted primarily as the loss of specific tumor-suppressive chromatin acetylation programs rather than as evidence that all acetylation is protective. For example, alterations affecting EP300/CREBBP may reduce acetyltransferase-dependent transcriptional regulation and thereby contribute to malignant progression. However, other acetylation-dependent modules may support tumor maintenance when they enhance oncogenic transcription, DNA damage adaptation, or survival signaling. Thus, the functional direction of histone acetylation in bladder cancer is determined by the affected genomic loci, the responsible acetylation regulator, and the molecular background of the tumor.

### 3.2. Non-Histone Acetylation Imbalance in Bladder Cancer

Evidence increasingly suggests that abnormal acetylation of non-histone proteins plays a major role in bladder cancer progression. Several transcription factors are subject to acetylation-dependent regulation, among which p53 and nuclear factor kappa B (NF-κB) are particularly noteworthy because of their well-established roles in cell-cycle control, apoptosis, and inflammatory signaling [86,87]. Disturbance of their acetylation status may compromise normal protein function and thereby favor tumor cell survival and proliferation. In addition, emerging studies point to an interplay between metabolic rewiring and non-histone acetylation in bladder cancer. One representative example is ACSL5, a key enzyme in fatty acid metabolism, whose overexpression alters acetyl-CoA availability and promotes 53BP1 acetylation. This modification activates the p53–p21 senescence axis and induces cellular senescence, suggesting that metabolic pathways can influence tumor behavior through regulation of protein acetylation [83]. Beyond p53, acetylation-dependent control of NF-κB is also highly relevant. The RelA/p65 subunit can be acetylated by p300/CBP at multiple lysine residues, thereby modulating its DNA-binding capacity, nuclear retention, and transcriptional output. Although many of these mechanisms have been characterized in other tumor types, persistent activation of NF-κB signaling is a recognized feature of bladder cancer and contributes to inflammation, tumor cell survival, and disease progression [88]. It is therefore plausible that disruption of acetylation-dependent NF-κB regulation represents one mechanism underlying its sustained activation in this malignancy.

The dual nature of acetylation is even more apparent for non-histone substrates. Acetylation of stress-response proteins such as p53 generally favors tumor-restraining outcomes, including cell-cycle arrest, senescence, or apoptosis. By contrast, acetylation-dependent regulation of signaling or cytoskeletal proteins may influence migration, invasion, or adaptive survival in a substrate-specific manner. Therefore, the functional interpretation of non-histone acetylation should be assigned at the level of individual substrates rather than at the level of acetylation as a broad modification class.

Overall, these observations indicate that non-histone acetylation adds an important layer of regulatory complexity to bladder cancer and broadens the range of potentially actionable acetylation-dependent vulnerabilities.

### 3.3. Functional Consequences of Dysregulated Acetylation in Bladder Cancer

The biological consequences of acetylation dysregulation in bladder cancer are broad and phenotypically consequential. By reshaping the activity of cell-cycle regulators, apoptotic mediators, EMT-associated programs, and treatment-response pathways, aberrant acetylation supports sustained growth, invasive progression, and diminished therapeutic sensitivity. These downstream effects highlight why acetylation should be viewed not only as a mechanistic layer of epigenetic regulation, but also as a determinant of clinically relevant tumor behavior.

#### 3.3.1. Proliferation and Cell Cycle Progression

Sustained proliferation and defective cell-cycle control are core features of malignant transformation [89,90]. In bladder cancer, these processes are shaped not only by canonical oncogenic signaling, but also by acetylation-dependent regulation of proteins that govern checkpoint control, cell-cycle progression, and proliferative transcriptional programs.

Consistent with this concept, aberrant acetylation contributes directly to bladder cancer cell proliferation and cell-cycle progression through modulation of key regulatory factors. Jia et al. demonstrated that p21 regulates HDAC2 phosphorylation through its interaction with CK2, thereby influencing the acetylation status of KLF4 [54]. Importantly, this acetylation-dependent change determines whether KLF4 acts as a cell-cycle suppressor or shifts toward a pro-proliferative role, ultimately affecting bladder cancer cell growth. Additional support for the importance of acetylation-related signaling comes from the study by Tan et al., who showed that the SIRT1 activator SRT1720 markedly inhibited the growth of both murine and human bladder cancer organoids, as well as murine in situ tumors and human patient-derived xenograft models [80]. Mechanistically, this suppressive effect was mediated through the SIRT1–hypoxia-inducible factor (HIF) axis, while mutation of Sirt1 enhanced organoid growth and reduced sensitivity to SRT1720, supporting SIRT1 as a functionally relevant target. These findings indicate that acetylation-associated signaling pathways actively contribute to the proliferative capacity of bladder cancer cells and may be therapeutically exploitable.

A comparable tumor-promoting role has been proposed for HDAC2, whose overexpression appears to shift the global acetylation landscape toward a more aggressive proliferative state. Consistent with this view, increased HDAC2 expression has been associated with enhanced proliferation, metastasis, and chemoresistance, underscoring the contribution of excessive deacetylation to malignant progression [91]. Beyond canonical acetylation regulators, acetyl-CoA-related metabolic enzymes may also participate in this process. For instance, ACAT1 has been reported to promote bladder cancer cell proliferation and metastasis through the AKT/GSK3β/c-Myc signaling pathway, at least in part by modulating cell-cycle progression and EMT-related programs [82]. This observation suggests that acetyl-associated metabolic reprogramming may cooperate with aberrant acetylation signaling to drive malignant progression. Taken together, current evidence supports a central role for acetylation imbalance in sustaining proliferative signaling and disrupting normal cell-cycle control in bladder cancer.

#### 3.3.2. Apoptosis and Cell Survival

Evasion of apoptosis is another major consequence of acetylation imbalance in bladder cancer. Rather than resulting from a single downstream lesion, this phenotype likely reflects the combined disruption of chromatin-based transcriptional control and non-histone acetylation-dependent regulation of survival pathways. Through these mechanisms, aberrant acetylation can weaken pro-apoptotic signaling while reinforcing tumor cell persistence under oncogenic and therapeutic stress [92,93,94].

A representative example is the SIRT1/p53 axis, in which deacetylation suppresses p53 activity and thereby blunts its pro-apoptotic output. In line with this mechanism, puerarin has been shown to induce apoptosis in T24 bladder cancer cells through the SIRT1/p53 pathway, accompanied by an increased Bax/Bcl-2 ratio, whereas pharmacological activation of SIRT1 partially reverses this effect [95]. Likewise, deacetylases such as SIRT7 appear to support tumor cell survival, since SIRT7 downregulation inhibits proliferation and enhances apoptosis in bladder cancer cells [85]. Further support comes from pharmacological studies showing that excessive deacetylase activity contributes to apoptotic resistance. For example, trichostatin A enhances gemcitabine-induced caspase-mediated apoptosis while suppressing NF-κB and AKT survival signaling, and newly developed HDAC inhibitor derivatives similarly exhibit apoptosis-associated antitumor effects both in vitro and in vivo [22]. Taken together, these findings suggest that acetylation imbalance raises the apoptotic threshold of bladder cancer cells by weakening pro-apoptotic signaling and reinforcing survival pathways, thereby promoting tumor persistence under both oncogenic and therapeutic stress. It should also be noted that the relative contribution of distinct acetylation-regulated apoptotic pathways may vary according to the molecular background of individual tumors, indicating that the impact of acetylation imbalance on cell death is context dependent.

#### 3.3.3. EMT, Metastasis and Therapeutic Resistance

Acetylation dysregulation is also linked to phenotypic plasticity, metastatic dissemination, and reduced therapeutic responsiveness in bladder cancer. These processes are closely interconnected: EMT promotes migratory and invasive behavior, enhances cellular adaptability, and often coincides with resistance to cytotoxic or immune-based therapies [96,97]. In this context, aberrant acetylation may facilitate aggressive disease behavior by coordinately reshaping chromatin states and non-histone protein function.

Mechanistically, altered histone acetylation can reprogram transcription of genes involved in epithelial identity, mesenchymal transition, and treatment response, whereas abnormal deacetylation of non-histone substrates can directly affect cytoskeletal remodeling, adhesion dynamics, and adaptive stress signaling [98]. Within this context, aberrant acetylation is likely to promote EMT-associated reprogramming through coordinated effects on chromatin architecture and non-histone protein function.

Altered histone acetylation can reshape the transcriptional landscape of genes involved in epithelial identity, mesenchymal transition, and treatment response by modulating chromatin accessibility. At the same time, dysregulated deacetylation of non-histone substrates may directly influence cytoskeletal remodeling, cell adhesion, migratory behavior, and adaptive stress signaling. Supporting this concept, HDAC6 and SIRT2 have been reported to promote bladder cancer cell migration and invasion by targeting the cytoskeletal protein cortactin, highlighting the importance of non-histone deacetylation in the regulation of cell motility [79]. Moreover, sirtuin deregulation may contribute to EMT-associated phenotypic plasticity, as SIRT7 downregulation has been linked to enhanced migration and invasion, reduced E-cadherin expression, and activation of the EZH2–CDH1 axis in bladder cancer [81]. Such EMT-related alterations may further foster therapeutic resistance by increasing cellular adaptability and survival under treatment pressure. Consistently, aberrant deacetylase activity has also been implicated in diminished treatment sensitivity; for example, HDAC7 restricts CD8+ T-cell tumor infiltration and limits immunotherapy sensitivity, whereas HDAC2 overexpression is associated with metastatic behavior and chemoresistance [23]. Overall, these observations indicate that acetylation imbalance promotes EMT-associated plasticity in bladder cancer, thereby facilitating metastatic progression and reducing therapeutic responsiveness.

Taken together, the current evidence supports a provisional context model for acetylation in bladder cancer. Acetylation tends to exert tumor-restraining effects when it preserves tumor-suppressive transcriptional programs or activates p53- and senescence-associated responses. Conversely, acetylation-related regulation may promote tumor progression when it stabilizes oncogenic signaling, enhances cytoskeletal plasticity, facilitates immune exclusion, or supports therapeutic adaptation. Importantly, direct comparative evidence across NMIBC versus MIBC and luminal-like versus basal-like bladder cancer remains limited. These distinctions should therefore be regarded as a working framework rather than a settled classification, and future studies should define acetylation dependencies in subtype-resolved models.

## 4. Natural Products Regulating Acetylation: Classification and Representative Compounds

**Figure 1:**
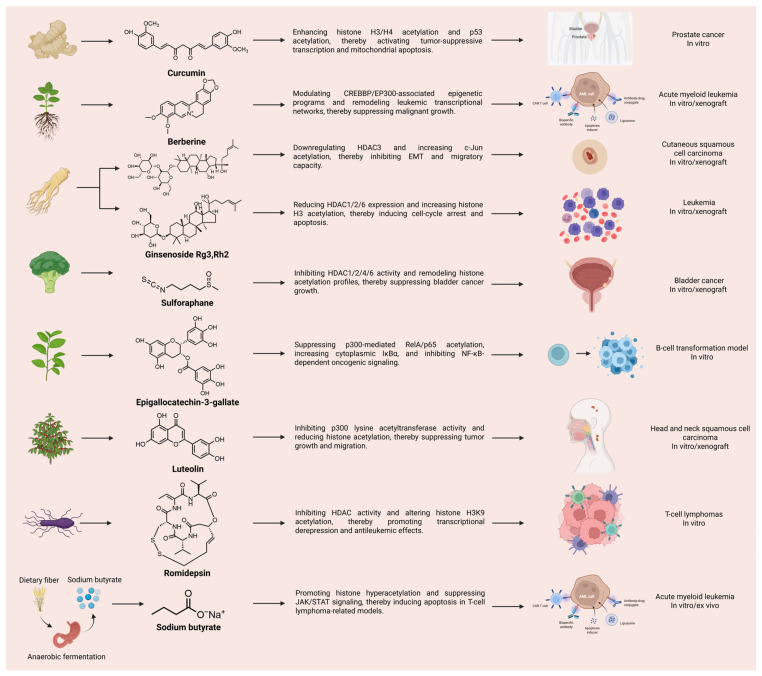
Representative natural products regulating acetylation across different cancer contexts.

Natural products influence acetylation through multiple mechanistic routes and across diverse cancer contexts. Rather than acting through a single uniform mode, these compounds may target acetylation-related enzymes, alter histone marks, or reshape the acetylation state of non-histone signaling proteins, thereby affecting proliferation, apoptosis, migration, EMT, and treatment response. Representative compounds and their associated cancer settings are summarized in Figure 1.

This section provides a concise cross-cancer overview of representative natural products reported to regulate acetylation. These studies are included to define the broader pharmacologic landscape and illustrate mechanistic possibilities, but they should be interpreted as contextual background rather than direct evidence for bladder cancer. Accordingly, compounds discussed here are not weighted equivalently to bladder cancer-based studies in the disease-specific synthesis presented in Section 5. Representative compounds and their associated cancer settings are summarized in Figure 1.

### 4.1. Curcumin

Curcumin is the major polyphenolic constituent isolated from Curcuma longa and is widely recognized for its anti-inflammatory, antioxidant, and antitumor properties. Increasing evidence suggests that curcumin can influence acetylation-related processes in a tumor-context-dependent manner. In prostate cancer, curcumin has been reported to increase histone H3/H4 acetylation and promote apoptosis in LNCaP cells [99]. In contrast, in glioma models, curcumin induces histone hypoacetylation together with caspase-3-dependent cell death [100]. These findings suggest that curcumin is capable of modulating histone acetylation-associated signaling, although the direction and biological consequences of this regulation appear to vary according to cellular context. Therefore, curcumin may be more appropriately regarded as a context-dependent modulator of acetylation-related pathways rather than a canonical direct acetylation-targeting agent.

### 4.2. Berberine

Berberine is an isoquinoline alkaloid derived from medicinal plants such as Coptis chinensis and has been reported to exert antiproliferative, pro-apoptotic, and cell cycle-arresting effects in multiple cancer models. Several studies suggest that berberine is linked to acetylation-related signaling in specific tumor settings. In acute myeloid leukemia, berberine was reported to modulate the expression of epigenetic regulators including CREBBP/EP300 and HDAC8, supporting its role as a putative epigenetic modulator. In addition, in breast cancer, berberine-derived compounds have shown p300 HAT-inhibitory activity, decreased H3K27 acetylation, and suppressed tumor growth and invasion, further suggesting that the berberine scaffold is compatible with acetylation-targeting anticancer strategies [101]. Nevertheless, for native berberine itself, direct validation as a canonical HAT-targeting anticancer agent remains limited.

### 4.3. Ginsenosides

Ginsenosides are representative bioactive constituents derived from Panax species and have been widely reported to possess anticancer properties. In melanoma, ginsenoside Rg3 suppresses tumor cell proliferation by downregulating HDAC3 and increasing p53 acetylation at K373/K382, thereby enhancing p53 transcriptional activity, inducing G0/G1 arrest, and inhibiting xenograft growth in vivo [69]. In cutaneous squamous cell carcinoma, Rg3 also exerts anti-metastatic activity through an acetylation-dependent mechanism by reducing HDAC3 expression and increasing c-Jun acetylation, which in turn suppresses epithelial–mesenchymal transition (EMT) and cell migration, as reflected by increased E-cadherin and reduced N-cadherin, vimentin, and Snail expression [102]. In addition to these non-histone acetylation-related effects, ginsenoside Rh2 has been reported to act as a natural HDAC inhibitor in leukemia, where it decreases HDAC1, HDAC2, and HDAC6 expression, enhances histone H3 acetylation and HAT activity, and induces G0/G1 arrest, apoptosis, and xenograft growth inhibition [103]. Collectively, these findings indicate that ginsenosides can modulate both histone and non-histone acetylation networks, thereby suppressing tumor progression through coordinated effects on proliferation, apoptosis, EMT, and tumor cell plasticity.

### 4.4. Sulforaphane

Sulforaphane (SFN), an isothiocyanate derived from cruciferous vegetables such as broccoli, is one of the best-characterized natural modulators of acetylation [104]. SFN is widely recognized as a natural HDAC inhibitor and has been shown in colorectal and prostate cancer models to suppress HDAC activity, increase histone H3/H4 acetylation, and upregulate tumor suppressor-associated molecules such as p21 and Bax. Importantly, in bladder cancer models, SFN and its analogue erucin have been demonstrated to inhibit HDAC1, HDAC2, HDAC4, and HDAC6 and to remodel histone acetylation profiles in both cultured cells and xenograft tumors [105]. Therefore, SFN currently represents one of the strongest examples of a natural product with relatively well-supported acetylation-targeting activity across multiple cancer settings, including bladder cancer.

### 4.5. Epigallocatechin-3-Gallate (EGCG)

EGCG is the most intensively studied catechin in green tea and is frequently cited as a natural inhibitor of acetylation-related signaling. Its best-defined actions in this context involve suppression of p300-dependent acetylation events linked to NF-κB activation, supporting the idea that natural compounds can also target non-histone acetylation-dependent transcriptional circuits [106].

### 4.6. Luteolin

Luteolin is a naturally occurring flavonoid widely found in plant-derived foods such as celery and parsley. Unlike many flavonoids that exhibit only broad and pleiotropic biological activities, luteolin has relatively direct mechanistic evidence linking it to acetylation regulation. In head and neck squamous cell carcinoma models, luteolin has been shown to inhibit p300 lysine acetyltransferase activity, reduce histone acetylation levels, and significantly suppress xenograft tumor growth [107].

### 4.7. Butyrate/Sodium Butyrate

Butyrate and its salt form sodium butyrate are naturally occurring short-chain fatty acids generated by anaerobic fermentation of dietary fiber in the gut and are among the best-known natural HDAC inhibitors. Butyrate and sodium butyrate are among the best-characterized natural HDAC inhibitors. In gastric cancer, sodium butyrate induces apoptosis and alters the expression of apoptosis-related genes, including upregulation of caspase-3 and DAPK1/2 with concomitant downregulation of Bcl-2 [108]. In colorectal cancer, sodium butyrate-induced histone hyperacetylation has been linked to growth inhibition, differentiation, and apoptosis [109]. Similar acetylation-associated antitumor effects have also been reported in acute myeloid leukemia, where butyrates increased histone H4 acetylation in primary blasts and promoted differentiation and apoptosis [110].

### 4.8. Romidepsin (FK228)

Romidepsin (FK228) is a natural product-derived HDAC inhibitor isolated from cultures of Chromobacterium violaceum and is one of the most clinically significant examples of acetylation-targeting natural agents. As a selective HDAC inhibitor, romidepsin has been approved for the treatment of certain T-cell lymphomas, providing strong clinical proof-of-concept that the strategy of “natural product-based HDAC inhibition–anticancer therapy” is not limited to preclinical investigation [111]. Beyond its clinical application, romidepsin has also been shown to induce cell cycle arrest, differentiation, and apoptosis, and to enhance antitumor effects when combined with agents such as cisplatin [112]. Therefore, romidepsin stands as a compelling example of successful clinical translation of natural products targeting acetylation.

Taken together, the natural products discussed in this section demonstrate that acetylation can be modulated through multiple pharmacological routes, including HDAC inhibition, HAT inhibition, sirtuin-associated regulation, and non-histone substrate acetylation. However, the strength of evidence differs substantially among compounds. Butyrate/sodium butyrate and romidepsin represent relatively mature HDAC-related examples, with romidepsin providing clinical proof-of-concept for natural product-derived HDAC inhibition in oncology. SFN has relatively strong mechanistic support as an HDAC-modulating dietary compound and also has direct relevance to bladder cancer, as discussed further in Section 5. By contrast, curcumin, berberine, EGCG, luteolin, and ginsenosides illustrate broader acetylation-related pharmacological possibilities, but much of their evidence derives from non-bladder cancer models and should therefore be interpreted as mechanistic background rather than direct BCa therapeutic evidence. This distinction is important because cross-cancer acetylation mechanisms may generate testable hypotheses, but disease-specific validation remains necessary before therapeutic conclusions can be drawn for bladder cancer.

## 5. Natural Products Targeting Acetylation: Implications for Bladder Cancer Therapy

Natural products have attracted sustained interest in cancer therapy because of their structural diversity, multitarget activity, and generally favorable tolerability profiles [113]. Within the field of epigenetic intervention, increasing evidence indicates that some natural compounds can suppress tumor progression by modulating acetylation-related enzymes or altering the acetylation status of histone and non-histone substrates [114]. However, for a review centered on bladder cancer, the key issue is not merely whether a compound modulates acetylation in oncology in general, but whether this relationship has been demonstrated directly in bladder cancer-relevant systems.

**Figure 2:**
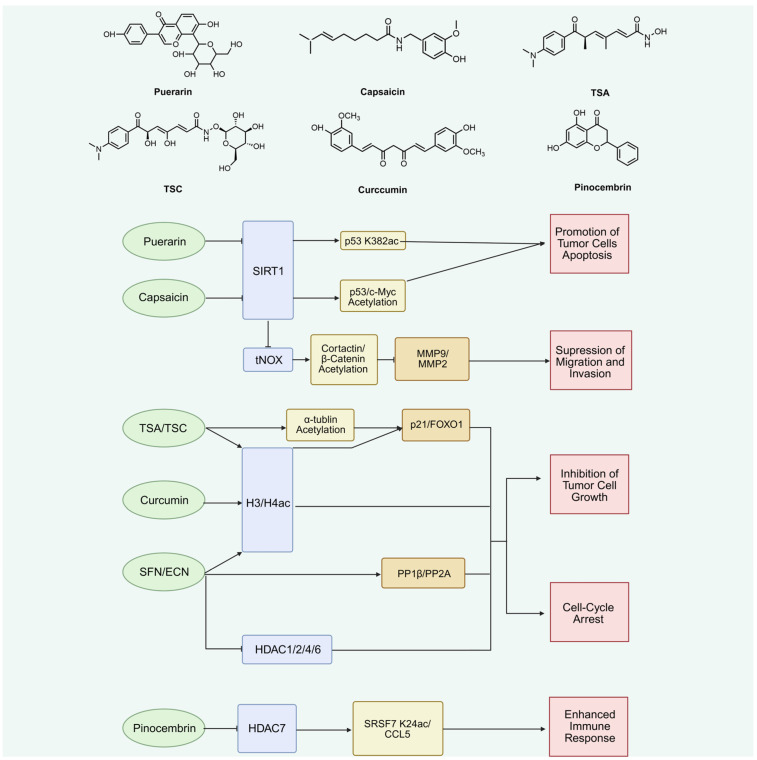
Acetylation-targeting natural products and their antitumor mechanisms in bladder cancer. Resveratrol is discussed in the text as pathway-informative evidence for SIRT1 signaling and is therefore not emphasized as a primary bladder cancer-directed candidate in the schematic.

In contrast to the cross-cancer overview provided above, this section focuses specifically on natural products for which bladder cancer-related evidence is available. Priority is given to studies performed in bladder cancer cell lines, xenograft models, organoids, immunocompetent models, or other BCa-relevant experimental systems. Evidence from other malignancies is used only to interpret broader mechanisms and is not considered sufficient to establish bladder cancer-specific therapeutic relevance. Under this framework, the currently available bladder cancer evidence clusters mainly around two tumor-intrinsic mechanisms—SIRT1-associated non-histone deacetylation and HDAC-dependent histone or cytoskeletal acetylation—and one emerging tumor microenvironment-related mechanism involving HDAC7-dependent immune regulation. Representative compounds discussed in this section are summarized in Figure 2.

Current evidence indicates that several natural products can influence bladder cancer biology through acetylation-related mechanisms, although the depth of mechanistic validation varies considerably among compounds. Figure 2 outlines the major acetylation-related mechanisms through which representative natural products may affect bladder cancer progression. However, these compounds differ markedly in target specificity, mechanistic completeness, and functional validation. To provide a clearer comparison, Table 3 maps each compound to its best-supported acetylation-linked node, downstream pathway or readout, and standardized biological outcome in bladder cancer models.

The bladder cancer-specific literature is still limited, but it is sufficiently developed to support a narrative synthesis organized by mechanism rather than by compound name alone. The first major module involves SIRT1-centered non-histone deacetylation. Puerarin has been reported to inhibit the SIRT1/p53 pathway in T24 bladder cancer cells, where treatment at 10, 50, and 100 μg/mL reduced cell viability and the 50 μg/mL concentration was selected for mechanistic assays [95]. This study connects puerarin with apoptosis induction, altered Bax/Bcl-2 balance, and suppression of proliferation, but its evidence is stronger for SIRT1/p53 pathway modulation than for a fully mapped lysine-specific acetylation event. Therefore, puerarin should be interpreted as an SIRT1-associated lead compound whose mechanistic plausibility requires further confirmation by direct target-engagement and site-specific p53 acetylation assays.

Capsaicin provides a more detailed non-histone acetylation model but also illustrates the importance of dose context. In bladder cancer cells, capsaicin at 100 and 200 μM suppresses tNOX/SIRT1 signaling, enhances acetylation of p53 or c-Myc, and reduces proliferative and survival phenotypes [115]. A separate migration-focused study further showed that capsaicin decreases SIRT1 expression and activity, increases cortactin and β-catenin acetylation, reduces MMP-2/MMP-9 activation, and impairs cell migration [116]. These findings form a relatively coherent compound–target–substrate–phenotype chain. Nevertheless, the active concentrations are high, and lower exposure can produce different biological outputs, including migration-promoting effects reported around 10 μM in some experimental settings. Capsaicin should therefore be positioned as a mechanistically informative SIRT1/tNOX probe rather than as a near-clinical candidate until bladder-specific pharmacokinetic and urothelial safety data are available.

Resveratrol occupies a different evidentiary position. In the TM4SF1-PPARγ-SIRT1 feedback loop, resveratrol was used as an SIRT1 activator to partially reverse apoptosis and cell-cycle effects caused by TM4SF1 knockdown [117]. This supports the functional relevance of SIRT1 signaling in bladder cancer, but it does not establish resveratrol itself as a validated acetylation-targeting therapy in this disease. Its value in the present review is therefore best understood as pathway-informative evidence demonstrating that SIRT1 activity can influence bladder cancer cell fate.

The second major module is HDAC-centered chromatin and cytoskeletal remodeling. Curcumin has been reported to increase histone H3/H4 acetylation and perturb cell-cycle and apoptotic programs in urothelial cancer cells under visible-light-enhanced conditions [118]. However, this experimental design narrows the generalizability of the finding. Curcumin should not be presented as an isoform-defined HDAC or HAT modulator in bladder cancer; rather, it represents a context-dependent acetylation-associated compound whose biological effect is influenced by photochemical conditions, formulation, and exposure. Trichostatin A (TSA) and trichostatin C (TSC) provide more classical HDAC-inhibitor comparators. TSA enhances gemcitabine-induced apoptosis and suppresses NF-κB/AKT survival signaling in bladder cancer cells [22], while another bladder cancer study showed that 125, 250, and 500 nM TSA caused cell-cycle arrest and intrinsic apoptosis, with the 24 h IC50 falling between 250 and 500 nM [119]. TSC increased histone H3 and α-tubulin acetylation, upregulated p21 and FOXO1, downregulated Axl, and inhibited bladder cancer growth with an IC50 of 4.16 μM in J82 cells [120]. These studies support HDAC inhibition as a therapeutically relevant acetylation axis, but they also raise the issue of selectivity because pan-HDAC inhibition may affect both tumor and normal urothelial programs.

Among dietary natural products, sulforaphane (SFN) and erucin (ECN) currently provide the most coherent bladder-oriented evidence. In bladder cancer models, SFN and ECN inhibited tumor cell growth with IC50 values of 5.66 ± 1.2 μM and 8.79 ± 1.3 μM, respectively, while normal urothelial cells were less sensitive [121]. A subsequent proteomic and histone-focused study showed that cruciferous isothiocyanates affect HDAC1, HDAC2, HDAC4, and HDAC6 and remodel histone acetylation- and phosphorylation-associated signaling in bladder cancer models [105]. This makes SFN/ECN one of the few examples in which potency, acetylation-linked mechanism, and bladder exposure logic point in the same direction. Even so, not every downstream event has been proven to result directly from HDAC inhibition, and the contribution of reactive oxygen species, metabolic stress, and broader proteomic remodeling must remain part of the interpretation.

The third module concerns acetylation-dependent immune regulation. Pinocembrin differs from the other compounds because its main value lies not in direct cytotoxicity but in reversal of immune exclusion. In bladder cancer, HDAC7 deacetylates SRSF7 at Lys24, thereby promoting BTRC-mediated SRSF7 degradation, impairing CCL5 mRNA processing and expression, reducing CD8-positive T-cell infiltration, and limiting response to PD-1 blockade. Pinocembrin was reported to counteract this HDAC7-driven axis, restore antitumor immune infiltration, and improve checkpoint blockade sensitivity in vivo [23]. This mechanism broadens the therapeutic meaning of acetylation targeting: the relevant endpoint may be immune reprogramming rather than direct tumor-cell killing. It also indicates that immunocompetent and orthotopic models will be essential for future evaluation.

The evidence summarized above indicates that acetylation-targeting natural products in bladder cancer should not be regarded as a uniform pharmacological category. Although several compounds have been reported to affect acetylation-related pathways, the depth of mechanistic validation varies considerably. A rigorous interpretation therefore requires distinguishing compounds with direct bladder cancer-specific mechanistic evidence from those supported mainly by cross-cancer observations, indirect pathway-level associations, or incomplete acetylation-site validation.

Among the compounds discussed in this review, SFN and ECN currently represent one of the strongest bladder cancer-specific examples. Their activity has been linked to HDAC1, HDAC2, HDAC4, and HDAC6 inhibition, histone-associated signaling changes, and growth suppression in bladder cancer models. This provides a relatively coherent connection between natural product exposure, HDAC modulation, acetylation remodeling, and antitumor phenotype. Nevertheless, these compounds should still be interpreted cautiously, because not all downstream effects have been definitively proven to result directly from HDAC inhibition.

Capsaicin provides a comparatively well-defined example of non-histone acetylation regulation. Its effects have been associated with the tNOX/SIRT1 axis, SIRT1 engagement, increased acetylation of p53, c-Myc, cortactin, and β-catenin, reduced MMP-2/MMP-9 activation, and suppression of migration-related phenotypes. This represents a more coherent compound–target–substrate–phenotype chain than many other natural products. However, the effective concentrations reported in bladder cancer models are relatively high, and dose-dependent complexity has been observed, indicating that its biological interpretation should remain context-aware.

Puerarin also supports the relevance of SIRT1-associated acetylation signaling in bladder cancer, particularly through the SIRT1/p53 axis. However, the evidence remains less complete because direct site-specific validation of p53 acetylation is limited, and most data are derived from cellular models. Similarly, curcumin has been associated with increased histone H3/H4 acetylation and apoptosis-related signaling, but the available bladder cancer evidence is condition-dependent and does not clearly define a specific HDAC or HAT target. These limitations suggest that puerarin and curcumin should be positioned as early-stage or context-dependent acetylation-associated modulators rather than definitive acetylation-targeting agents in bladder cancer.

TSA and TSC provide useful pharmacological benchmarks for HDAC-dependent vulnerability. Their ability to induce histone and α-tubulin acetylation, cell-cycle arrest, and apoptosis supports the therapeutic relevance of HDAC inhibition in bladder cancer models. However, these agents are broad HDAC inhibitors rather than bladder cancer-selective natural products, and their value lies mainly in validating HDAC-dependent acetylation remodeling as a biologically relevant mechanism. Pinocembrin is distinct from these tumor-intrinsic examples because its primary significance lies in immune reprogramming. By modulating the HDAC7–SRSF7–CCL5 axis, pinocembrin links acetylation regulation to CD8-positive T-cell infiltration and improved sensitivity to PD-1 blockade, thereby expanding the conceptual scope of acetylation-targeting natural products from direct tumor-cell suppression to tumor immune microenvironment remodeling.

Taken together, the current literature supports an evidence-weighted hierarchy rather than a uniform class effect. SFN/ECN have the strongest dietary and bladder-oriented mechanistic rationale; capsaicin and puerarin illustrate SIRT1-associated non-histone acetylation mechanisms; TSA/TSC serve as pharmacological HDAC-inhibition benchmarks; curcumin remains condition-dependent and mechanistically unresolved; and pinocembrin represents an emerging immune-acetylation strategy. This stratification provides a more critical framework than a descriptive list of compounds and clarifies the main contribution of this review: natural products targeting acetylation in bladder cancer should be evaluated according to disease specificity, mechanistic completeness, acetylation-event resolution, and functional relevance.

## 6. Translational Challenges and Future Perspectives

### 6.1. Comparative Translational Framework: Potency, Selectivity, and Therapeutic Relevance

Although natural products targeting acetylation provide a mechanistically attractive strategy for bladder cancer intervention, their translational relevance cannot be inferred solely from in vitro modulation of HDAC activity, histone acetylation, sirtuin signaling, or non-histone substrate acetylation. A clinically meaningful candidate should be evaluated according to at least four criteria: quantitative potency in bladder cancer models, acetylation-target selectivity or mechanistic resolution, in vivo or pharmacokinetic support, and therapeutic feasibility under clinically realistic exposure conditions. This comparative framework is particularly important in bladder cancer, where urinary exposure and intravesical delivery may create opportunities distinct from systemic solid tumor therapy [122].

Among the compounds discussed in this review, sulforaphane (SFN) and erucin (ECN) currently occupy the most favorable translational position. In bladder cancer models, SFN and ECN showed IC_50_ values of 5.66 ± 1.2 μM and 8.79 ± 1.3 μM, respectively, and normal cells were reported to be less sensitive, suggesting a degree of tumor selectivity at the cellular level [121]. These compounds are also mechanistically linked to HDAC inhibition and histone-associated signaling changes in bladder cancer [105].

Capsaicin and puerarin are mechanistically informative but less mature from a translational standpoint. Capsaicin has been shown to affect tNOX/SIRT1-related signaling in bladder cancer cells, with 100 and 200 μM reducing SIRT1 expression, increasing p53 or c-Myc acetylation, and suppressing migration-related signaling [123]. A separate study further showed that capsaicin can engage SIRT1 and enhance acetylation of cortactin and β-catenin, thereby reducing MMP-2/MMP-9 activation and impairing cell migration [115]. However, the relatively high effective concentration range, together with reports that 10 μM capsaicin may increase migration in some bladder cancer settings, indicates that the exposure window and context-dependent effects require careful evaluation before clinical interpretation [115]. 

Classical HDAC inhibitors of natural origin or natural-product-derived scaffolds, such as trichostatin A (TSA), trichostatin C (TSC), and romidepsin, provide important pharmacological reference points but raise different translational concerns. TSA has been reported to induce bladder cancer cell-cycle arrest and apoptosis at 125–500 nM, demonstrating strong cellular potency [119]. TSC increases acetylated histone H3 and acetylated α-tubulin, consistent with inhibition of HDAC1- and HDAC6-related signaling [118]. Nevertheless, potent broad-spectrum HDAC inhibition is not automatically advantageous for bladder cancer therapy because systemic toxicity, isoform non-selectivity, and effects on normal immune or urothelial cells may limit therapeutic windows. Romidepsin further demonstrates that natural-product-derived HDAC inhibitors can achieve regulatory approval, but its approved indication is cutaneous T-cell lymphoma rather than bladder cancer [124,125]. Therefore, romidepsin is best interpreted as a regulatory proof-of-concept for epigenetic drug development, not as direct evidence for bladder cancer therapy.

A distinct translational opportunity is emerging from immune-acetylation biology. Pinocembrin has been identified as a selective HDAC7 inhibitor in bladder cancer, and HDAC7 inhibition restored CD8-positive T-cell infiltration and improved PD-1 antibody efficacy in preclinical models. This finding shifts the translational endpoint from direct cytotoxicity to immune reprogramming. Accordingly, compounds acting through this axis should be evaluated in immunocompetent or humanized bladder cancer models, with predefined immune endpoints such as CCL5 expression, CD8-positive T-cell recruitment, IFN-γ/granzyme B activity, and checkpoint blockade sensitivity [23].

To further clarify the translational relevance of these acetylation-targeting natural products, Table 4 summarizes the available evidence regarding bladder cancer-specific potency, mechanistic validation, in vivo efficacy, pharmacokinetic features, and major translational limitations.

### 6.2. Clinical Applicability: Current Standards, Translation Barriers, and HDAC-Inhibitor Comparisons

The clinical applicability of acetylation-targeting natural products should be interpreted in relation to the current bladder cancer treatment landscape. For NMIBC, standard management remains centered on transurethral resection of bladder tumor (TURBT), followed by risk-adapted intravesical therapy, including intravesical chemotherapy or BCG-based immunotherapy [128,129]. For BCG-unresponsive high-risk NMIBC, pembrolizumab and nogapendekin alfa inbakicept-pmln plus BCG have been approved for selected patients [130]. For locally advanced or metastatic urothelial carcinoma, enfortumab vedotin plus pembrolizumab has become an approved systemic treatment option. Against this background, acetylation-targeting natural products should not be positioned as replacements for established therapies, but rather as investigational adjuncts, bladder-directed chemopreventive candidates, intravesical formulation candidates, or immunotherapy-sensitizing agents. Comparison with clinically used HDAC inhibitors also supports a cautious interpretation. Romidepsin demonstrates that natural product-derived HDAC inhibition can achieve regulatory approval in oncology, but its approved indications are T-cell lymphomas rather than bladder cancer [124,125]. Moreover, a phase II study of vorinostat in advanced urothelial carcinoma reported limited efficacy and significant toxicity, indicating that broad HDAC inhibition alone is unlikely to be sufficient for bladder cancer translation [131].

Although Table 4 provides a comparative overview of potency and mechanism maturity, clinical translation requires additional consideration of exposure, safety, formulation, and regulatory feasibility. A natural product with clear acetylation-modulating activity in vitro may still have limited therapeutic value if the active concentration cannot be achieved in the bladder, if the safety window in normal urothelium is undefined, or if the preparation lacks reproducible composition and pharmacodynamic consistency. Therefore, the translational interpretation of acetylation-targeting natural products should not rely solely on cytotoxicity or pathway modulation, but should integrate pharmacokinetic accessibility, route of administration, and clinical development feasibility.

In this regard, SFN/ECN appear comparatively favorable because bladder exposure is pharmacologically plausible. Sulforaphane is rapidly eliminated through urinary excretion, and urinary concentrations of sulforaphane equivalents have been reported to be 2–4 orders of magnitude higher than plasma concentrations, with bladder tissue showing relatively high uptake among genitourinary organs [132]. This feature provides a rationale for bladder-directed chemoprevention or intravesical development, particularly in NMIBC [133]. However, this exposure advantage should not be interpreted as clinical efficacy. The registered bladder cancer sulforaphane study NCT03517995 was withdrawn before efficacy data were available, indicating that clinical validation remains incomplete.

Curcumin illustrates the opposite situation, in which broad mechanistic activity is constrained by poor systemic exposure. Although curcumin has been associated with histone H3/H4 acetylation and apoptosis-related signaling in urothelial cancer cells, clinical pharmacokinetic data show that oral administration of 8 g/day produces plasma levels of only approximately 22–41 ng/mL [117,134]. This marked exposure limitation suggests that curcumin should be discussed as a formulation-dependent or local-delivery-dependent candidate rather than as a readily translatable systemic acetylation-targeting agent.

Safety and formulation issues also require more explicit consideration. Natural origin should not be equated with low toxicity. Compounds that require high micromolar or microgram-per-milliliter concentrations in vitro, such as capsaicin and puerarin, require evaluation of normal urothelial toxicity, mucosal irritation, and repeated-dose tolerability before therapeutic interpretation. Similarly, broad HDAC inhibitors such as TSA/TSC may demonstrate strong acetylation-related activity, but their lack of isoform selectivity raises concerns regarding effects on normal epithelial and immune compartments [135,136,137]. For these reasons, bladder-oriented delivery systems, including intravesical nanoparticles, liposomes, mucoadhesive hydrogels, or sustained-release formulations, may be necessary to improve local exposure while limiting systemic toxicity [138,139].

Finally, regulatory feasibility should be incorporated into the evaluation of natural products. Botanical or natural-product-derived agents intended for therapeutic use require defined composition, batch-to-batch consistency, stability testing, pharmacokinetic characterization, safety evaluation, and indication-specific clinical evidence [140,141]. The FDA guidance on botanical drug development emphasizes that botanical products submitted through IND or NDA pathways must meet appropriate standards for development, manufacturing, and clinical evidence. Thus, natural origin may facilitate discovery, but it does not reduce the evidentiary threshold required for drug development.

### 6.3. Future Research Priorities: Selective Modulation, Bladder-Specific Delivery, and Immunotherapy Integration

Based on the comparative and translational considerations discussed above, future studies should move from broad acetylation modulation toward more selective, mechanism-defined, and bladder-oriented development strategies. First, compounds with similar mechanistic labels should not be considered therapeutically equivalent. The term “acetylation-targeting” covers substantially different pharmacological situations, including broad HDAC inhibition, SIRT1-associated non-histone deacetylation control, histone acetylation remodeling, cytoskeletal substrate acetylation, and immune-acetylation reprogramming. Second, the strongest translational candidates are not necessarily those with the broadest biological effects, but those with the most coherent alignment among target engagement, acetylation remodeling, potency, exposure, and disease context. From this perspective, SFN/ECN currently show the strongest bladder-oriented rationale, whereas capsaicin, puerarin, and curcumin remain limited by exposure, formulation, or safety-window uncertainties. Third, immune-acetylation modulation, represented by the HDAC7/SRSF7/CCL5 axis, provides a distinct therapeutic direction that should be evaluated using immune-specific endpoints rather than conventional cytotoxicity alone.

Future development should therefore move from broad acetylation modulation toward selective and mechanism-defined acetylation intervention. A priority is to identify natural products or natural product-derived analogues with clearer selectivity for disease-relevant HDAC, HAT, or sirtuin isoforms [142]. This is particularly important because pan-HDAC inhibition or poorly defined acetylation modulation may affect normal urothelial, stromal, or immune compartments as well as tumor cells. Candidate compounds should be screened using HDAC/HAT/sirtuin isoform panels, direct target-engagement assays, and site-specific acetylome profiling [143,144]. These approaches would help distinguish compounds that directly remodel therapeutically relevant acetylation events from those that alter acetylation indirectly through stress responses, metabolic disruption, or nonspecific cytotoxicity.

A second priority is the development of bladder cancer-specific delivery strategies. Bladder cancer, especially NMIBC, offers a unique therapeutic window because the urothelium is directly accessible through intravesical administration [122]. This route may allow local concentrations that are difficult to achieve systemically while reducing systemic toxicity. Therefore, acetylation-targeting natural products with poor oral bioavailability or limited systemic exposure should not be excluded solely on pharmacokinetic grounds; instead, they should be evaluated in bladder-oriented delivery systems. Intravesical nanoparticles, liposomes, mucoadhesive hydrogels, sustained-release depots, and permeability-optimized formulations may improve urinary stability, urothelial retention, tissue penetration, and repeated-dose feasibility [138,139]. However, formulation development should not be assessed only by encapsulation efficiency or release kinetics. More relevant endpoints include retention after voiding, depth of urothelial penetration, preservation of normal barrier integrity, local inflammatory response, compatibility with repeated instillation, and maintenance of acetylation-related pharmacodynamic effects in vivo.

A third priority is to integrate acetylation-targeting strategies with immunotherapy-based treatment frameworks. The HDAC7/SRSF7/CCL5 axis suggests that acetylation regulation can reshape antitumor immunity by influencing chemokine processing and CD8-positive T-cell infiltration [23]. This expands the therapeutic relevance of acetylation-targeting natural products beyond direct tumor-cell growth inhibition. Future studies should therefore evaluate whether selected acetylation modulators can convert immune-excluded or immune-cold bladder tumors into more inflamed and checkpoint-sensitive states [145]. For this purpose, immunocompetent, orthotopic, or humanized bladder cancer models will be more informative than conventional tumor-cell viability assays. Key endpoints should include CCL5 expression, CD8-positive T-cell recruitment, IFN-γ and granzyme B activity, antigen-presentation markers, and response to PD-1/PD-L1 blockade.

Safety and regulatory readiness should be incorporated into this development pathway from the early stage. Natural origin should not be equated with low toxicity or simplified clinical development. Compounds requiring high micromolar or microgram-per-milliliter concentrations must be evaluated for normal urothelial toxicity, mucosal irritation, inflammatory response, and repeated-dose tolerability. Broad HDAC inhibitors require additional caution because their epigenetic effects may extend beyond tumor cells to normal epithelial and immune compartments [135]. From a regulatory perspective, natural products intended as therapeutic agents still require defined composition, reproducible manufacturing, stability, pharmacokinetic characterization, safety evaluation, and indication-specific clinical evidence. For botanical or dietary-derived preparations, batch consistency [140], active-marker definition [141], and mechanism-linked potency assays are particularly important [146]. Romidepsin provides a useful precedent showing that natural product-derived HDAC inhibitors can achieve regulatory approval, but its approved indications are hematologic malignancies rather than bladder cancer; therefore, it should be viewed as a proof-of-concept for epigenetic drug development, not as direct bladder cancer evidence [124].

Future studies should follow a staged and subtype-aware development strategy. The first stage should establish mechanistic precision by confirming direct target engagement, defining isoform selectivity, and mapping site-specific histone or non-histone acetylation events. The second stage should perform exposure-matched validation by comparing active concentrations observed in vitro with achievable levels in plasma, urine, bladder tissue, and tumor tissue under realistic routes of administration. The third stage should optimize bladder-specific delivery and safety, particularly for repeated intravesical administration. The fourth stage should test rational combinations, especially integration with immune checkpoint blockade when acetylation modulation affects chemokine expression, immune infiltration, or T-cell activity. Finally, candidate compounds should be evaluated in subtype-aware models that distinguish NMIBC from MIBC, luminal-like from basal-like tumors, and treatment-naive from therapy-resistant states [147,148].

Overall, the clinical translation of natural products targeting acetylation in bladder cancer remains promising but immature. The field should move beyond simply expanding the compound list and instead prioritize candidates with coherent target engagement, measurable acetylation remodeling, bladder-relevant exposure, defined safety windows, and clinically meaningful functional outcomes. In this framework, SFN/ECN represent the most rational dietary candidates for bladder-directed development, TSA/TSC serve as potent pharmacological references, pinocembrin highlights an emerging immunotherapy-sensitizing strategy, and curcumin, capsaicin, and puerarin remain valuable but less mature mechanistic leads. This staged and evidence-weighted approach may provide a more realistic path toward translating acetylation-modulating natural products into bladder cancer therapy.

## 7. Conclusions

The present review highlights acetylation as a context-dependent regulatory layer in bladder cancer rather than a uniformly oncogenic or tumor-suppressive modification. Its biological consequence depends on the specific acetylation regulator involved, the substrate being modified, the affected lysine residue, and the molecular background of the tumor. This point is particularly relevant for bladder cancer, which exhibits marked heterogeneity between NMIBC and MIBC, as well as among luminal-like, basal-like, immune-infiltrated, and therapy-resistant states. Therefore, acetylation should not be interpreted simply as a global increase or decrease in acetylation levels, but rather as a set of discrete regulatory events that influence tumor behavior in a substrate- and context-specific manner.

Current evidence suggests that acetylation dysregulation contributes to bladder cancer progression through several functional routes. Altered histone acetylation can reshape chromatin accessibility and transcriptional programs, thereby influencing tumor-suppressor gene expression, cell-cycle regulation, and apoptosis. In parallel, non-histone acetylation affects the activity, stability, and localization of key signaling proteins, including p53, cytoskeletal regulators, and immune-related factors. These mechanisms link acetylation to proliferation, apoptosis resistance, migration, invasion, immune evasion, and treatment response. However, the available data also indicate that the direction of effect is highly dependent on the molecular setting. For example, restoration of p53 acetylation may enhance tumor-suppressive signaling, whereas acetylation-dependent regulation of cytoskeletal or immune-modulatory pathways may support invasion or alter antitumor immunity.

Natural products provide a useful but heterogeneous source of acetylation-modulating agents. The compounds discussed in this review do not represent a uniform pharmacological class. Instead, they differ in disease specificity, target engagement, acetylation-site resolution, and functional validation. In bladder cancer, the most coherent evidence currently clusters around three mechanistic axes: SIRT1-associated non-histone deacetylation, HDAC-centered histone or cytoskeletal acetylation, and immune-acetylation regulation. Puerarin and capsaicin mainly illustrate SIRT1-related control of p53 activity or migration-associated substrates; SFN/ECN and TSA/TSC support the relevance of HDAC-dependent acetylation remodeling; and pinocembrin highlights a distinct immunological mechanism involving the HDAC7/SRSF7/CCL5 axis. These findings suggest that natural products may affect bladder cancer not only by directly suppressing tumor-cell growth, but also by modulating migration, stress responses, and immune infiltration.

Nevertheless, the strength of evidence varies considerably among compounds. SFN/ECN currently have relatively strong bladder cancer-specific support because their effects are linked to HDAC inhibition, histone-associated signaling changes, and quantitative activity in bladder cancer models. Capsaicin provides a more defined non-histone acetylation mechanism through SIRT1 and cytoskeletal substrates, but its concentration dependence and context-specific effects require cautious interpretation. Pinocembrin is particularly notable because it connects acetylation regulation with immune checkpoint responsiveness rather than conventional cytotoxicity. By contrast, several other natural products are better viewed as mechanistic references or early-stage candidates unless additional bladder cancer-specific validation is provided. This distinction is important because evidence from other malignancies can support mechanistic plausibility, but it cannot substitute for direct validation in bladder cancer.

A major limitation of the current field is that many studies remain descriptive. Changes in HDAC expression, sirtuin activity, or global acetylation markers are often reported without precise mapping of the acetylated substrate or lysine site responsible for the phenotype. In addition, direct target engagement is not always demonstrated, and many studies rely on established cell lines rather than patient-derived organoids, orthotopic models, immunocompetent systems, or therapy-resistant models. These limitations make it difficult to determine whether a compound acts through a defined acetylation-dependent mechanism or through broader stress, metabolic, or transcriptional effects.

Future studies should therefore move from association-based observations toward mechanism-resolved validation. Priority should be given to identifying direct acetylation targets, mapping site-specific histone and non-histone acetylation changes, and determining whether these events are functionally required for the observed antitumor phenotype. Equally important, acetylation-targeting strategies should be tested in subtype-aware models that distinguish NMIBC from MIBC, luminal-like from basal-like tumors, and treatment-naive from therapy-resistant disease. Such studies would help define which acetylation circuits are therapeutically exploitable in specific bladder cancer contexts.

Overall, this review supports an evidence-weighted framework for understanding natural products targeting acetylation in bladder cancer. Rather than treating these compounds as a broad group of low-toxicity epigenetic modulators, they should be stratified according to bladder cancer-specific evidence, target specificity, acetylation-site resolution, functional consequence, and therapeutic context. Acetylation represents a dynamic interface connecting chromatin regulation, protein function, immune response, and treatment sensitivity. Natural products may serve both as candidate therapeutic agents and as biological probes for identifying acetylation-dependent vulnerabilities. Advancing this field will require more rigorous mechanistic studies, stronger bladder cancer-specific validation, and better integration with molecular subtype and treatment-response contexts.

## Figures and Tables

**Table 1:** Representative acetylation-related regulators, molecular mechanisms, and biological consequences across different cancer contexts.

Regulator	Direct Substrate	Mechanism	Consequence	Cancer Context	Reference
EP300	HAT domain	Reduced HAT activity impairs tumor-suppressive transcription	Promotes tumor progression	Bladder cancer	[13]
HAT1	H2AK5ac	Catalyzes H2AK5 acetylation to support growth-related transcription	Promotes proliferation	HeLa cells	[25]
hMOF	Elevated H4K16ac	Increases H4K16ac and facilitates S-phase entry	Promotes proliferation	NSCLC	[26]
HDAC2	H3K27ac	Elevates H3K27ac at the H19 locus and activates the H19/MMP14 axis	Promotes invasion and metastasis	Colorectal cancer	[27]
HDAC1	p21-associated chromatin	Represses p21 transcription through chromatin deacetylation	Influences cell-cycle arrest and apoptosis	Gastric cancer	[28]
HDAC8	AKT1 K426	Deacetylates AKT1 and enhances AKT signaling	Promotes invasion and metastasis	Breast cancer	[29]
HDAC10	AKT1	Enhances AKT activation	Promotes proliferation	Lung cancer	[30]
KAT6A	H3K23ac	Acetylates H3K23 and enhances PIK3CA transcription via TRIM24 recruitment	Activates survival signaling	Glioblastoma	[31]
NAT10	BCL-XL mRNA ac4C	Acetylates and stabilizes BCL-XL mRNA, reinforcing PI3K–AKT signaling	Enhances cell survival	Multiple myeloma	[32]
SIRT1	Akt	Induces SIRT1-dependent AKT deacetylation and rewires AKT/mTOR signaling	Alters survival signaling	Pleural mesothelioma	[33]
TIP60	p53 K120	Acetylates p53 at K120 and shifts p53 transcriptional output	Influences cell-cycle arrest and apoptosis	Colorectal cancer/Lung cancer cells	[34]
TIP60	E2F1	Acetylates E2F1 and upregulates ERCC1	Promotes DNA repair and chemoresistance	Lung adenocarcinoma	[35]
PP2Cδ	p53 acetylation machinery	Suppresses p300-mediated p53 acetylation via ATM/BRCA1 signaling	Attenuates apoptosis	Breast cancer	[36]
Smad1	p53	Reduces p53 acetylation by sequestering p300 while enhancing Smad1 acetylation	Promotes therapy resistance	Gliomas	[37]

**Table 2:** Representative acetylation-related regulators, underlying mechanisms, and functional significance in bladder cancer.

Regulator	Class	Mechanism/Target	Functional Consequence	Evidence Level	Reference
EP300	HAT-encoding gene	HAT-domain missense mutations	Promoting proliferation and tumor progression	Genomic analysis	[13]
KAT8	HAT	YEATS4	Sustaining DNA damage repair and promoting therapeutic resistance	In vivo/In vitro	[78]
HDAC2	HDAC	KLF4	Promoting proliferation, cell cycle progression, metastasis, chemoresistance	In vitro	[54]
HDAC6	HDAC	Cortactin	Promoting migration, invasion and metastasis	In vitro	[79]
HDAC7	HDAC	SRSF7	Reducing immunotherapy sensitivity and Promoting immune evasion	In vivo/In vitro	[23]
SIRT1	Sirtuin	p53/FOXO3a/Beclin1/HIF-1α	Modulating proliferation, stress adaptation and autophagy	In vivo/In vitro	[80]
SIRT2	Sirtuin	Cortactin	Promoting migration, invasion and metastasis	In vitro	[79]
SIRT7	Sirtuin	EZH2-CDH1 axis	Suppressing apoptosis; promoting EMT, migration, and invasion	In vitro	[81]
ACAT1	Acetyl-associated metabolic enzyme	AKT/GSK3β/c-Myc	Promoting proliferation, cell cycle progression, EMT and metastasis	In vivo/In vitro	[82]
ACSL5	Acetyl-associated metabolic enzyme	53BP1	Inducing senescence; suppressing tumor cell growth	In vitro	[83]

**Table 3:** Mechanistic mapping of representative natural products to acetylation-linked targets and functional outcomes in bladder cancer.

Compound	Acetylation-Linked Node	Downstream Pathway/Readout	Functional Outcome	Reference
Puerarin	SIRT1/p53 axis; proposed p53 acetylation restoration	Bax/Bcl-2 shift; apoptosis signaling	Suppresses proliferation; induces apoptosis	[95]
Capsaicin	tNOX/SIRT1 axis; p53/c-Myc, cortactin and β-catenin acetylation	MMP-2/MMP-9 reduction; cytoskeletal remodeling	Suppresses migration/invasion; induces apoptosis	[115]
Resveratrol	SIRT1 activation as pathway intervention	TM4SF1–PPARγ–SIRT1 feedback loop	Supports SIRT1 pathway relevance	[116]
Curcumin	Histone H3/H4 acetylation under visible-light-enhanced conditions	Cell-cycle and apoptosis-related signaling	Induces cell-cycle perturbation; induces apoptosis	[117]
TSA/TSC	HDAC inhibition; histone H3 and α-tubulin acetylation	NF-κB/AKT, p21, FOXO1 and Axl-related signaling	Induces cell-cycle arrest; induces apoptosis; suppresses proliferation	[22,118]
SFN/ECN	HDAC1/2/4/6-associated histone remodeling	Histone acetylation/phosphorylation changes	Suppresses proliferation; induces cell-cycle arrest	[105]
Pinocembrin	HDAC7–SRSF7 Lys24 acetylation axis	SRSF7 stability; CCL5 processing; CD8-positive T-cell infiltration	Enhances immune infiltration; improves immunotherapy sensitivity	[23]

**Table 4:** Comparative translational positioning of representative acetylation-targeting natural products in bladder cancer.

Compound	Quantitative Potency	Acetylation Mechanism	In vivo, Pharmacokinetic, or Clinical Support	Translational Positioning	Reference
SFN/ECN	SFN: IC_50_ = 5.66 ± 1.2 μM; ECN: IC_50_ = 8.79 ± 1.3 μM	Associated with HDAC1/2/4/6 inhibition and histone remodeling	Strongest bladder-exposure support, with xenograft and urinary-exposure evidence	Most favorable dietary candidate group for bladder-oriented development	[105,121]
Capsaicin	Acetylation-related effects mostly at 100–200 μM	Linked to tNOX/SIRT1 signaling and acetylation of p53/c-Myc, cortactin, and β-catenin	Limited translational support, with no mature BCa-specific PK or clinical evidence	Non-histone acetylation mechanistic lead	[115,123]
Puerarin	Tested at 10, 50, and 100 μg/mL in T24 cells; 50 μg/mL used for mechanism assays	SIRT1/p53 pathway implicated	Limited translational support, with insufficient BCa-specific in vivo	Early-stage SIRT1/p53-related candidate	[126]
TSA	Active at 125–500 nM in BCa cells	Broad HDAC inhibition	Preclinical BCa support, but systemic translation is limited by toxicity	Potent pharmacological benchmark	[119,127]
Pinocembrin	Immune-related activity rather than classical IC_50_-centered cytotoxicity	HDAC7–SRSF7 Lys24–CCL5 axis	Preclinical immune-efficacy support, including enhanced CD8-positive T-cell infiltration and PD-1 response	Immunotherapy-sensitizing candidate	[23]

## Data Availability

No new data were created or analyzed in this study. Data sharing is not applicable to this article.

## Abbreviations

The following abbreviations are used in this manuscript:

BCa	Bladder cancer
NMIBC	Non-muscle-invasive bladder cancer
MIBC	Muscle-invasive bladder cancer
HAT	Histone acetyltransferase
HDAC	Histone deacetylase
HDACis	Histone deacetylase inhibitors
PCAF	p300/CBP-associated factor
AKT	Protein kinase B
PI3K	Phosphatidylinositol 3-kinase
PI3K/AKT/mTOR	Phosphatidylinositol 3-kinase/protein kinase B/mechanistic target of rapamycin
MAPK	Mitogen-activated protein kinase
PIP3	Phosphatidylinositol (3,4,5)-trisphosphate
RAF	Rapidly accelerated fibrosarcoma
MEK	MAPK/ERK kinase
ERK	Extracellular signal-regulated kinase
EGFR	Epidermal growth factor receptor
RB	Retinoblastoma protein
NF-κB	Nuclear factor kappa B
EMT	Epithelial–mesenchymal transition
NSCLC	Non-small-cell lung cancer
CD8+	Cluster of differentiation 8-positive
PD-L1	Programmed death-ligand 1
PD-1	Programmed cell death protein 1
HIF	Hypoxia-inducible factor
HIF-1α	Hypoxia-inducible factor-1 alpha
RNA	Ribonucleic acid
RNAi	RNA interference
DNA	Deoxyribonucleic acid
EP300	E1A binding protein p300
CREBBP/CBP	CREB-binding protein
KDM6A	Lysine demethylase 6A
ARID1A	AT-rich interaction domain 1A
hMOF	Human males absent on the first
KAT6A	Lysine acetyltransferase 6A
KAT8	Lysine acetyltransferase 8
TIP60	Tat-interactive protein, 60 kDa
GCN5	General control non-derepressible 5
SIRT	Sirtuin
AKT1	AKT serine/threonine kinase 1
PIK3CA	Phosphatidylinositol-4,5-bisphosphate 3-kinase catalytic subunit alpha
NAT10	N-acetyltransferase 10
PARP1	Poly(ADP-ribose) polymerase 1
ERCC1	Excision repair cross-complementation group 1
E2F1	E2F transcription factor 1
PP2Cδ	Protein phosphatase 2C delta
ATM	Ataxia telangiectasia mutated
BRCA1	Breast cancer susceptibility gene 1
KLF4	Kruppel-like factor 4
CK2	Casein kinase 2
ACAT1	Acetyl-CoA acetyltransferase 1
ACSL5	Acyl-CoA synthetase long-chain family member 5
53BP1	p53-binding protein 1
p53	Tumor protein p53
p21	Cyclin-dependent kinase inhibitor 1A
p73	Tumor protein p73
FOXO1	Forkhead box O1
FOXO3a	Forkhead box O3a
c-Myc	MYC proto-oncogene protein
c-Jun	Jun proto-oncogene protein
BCL-XL	B-cell lymphoma-extra large/BCL2-like 1
Bax	BCL2-associated X protein
Bcl-2	B-cell lymphoma 2
SRSF7	Serine/arginine-rich splicing factor 7
BTRC	Beta-transducin repeat containing E3 ubiquitin protein ligase
CCL5	C-C motif chemokine ligand 5
TM4SF1	Transmembrane 4 L six family member 1
PPARγ	Peroxisome proliferator-activated receptor gamma
GSK3β	Glycogen synthase kinase 3 beta
TRIM24	Tripartite motif containing 24
YEATS4	YEATS domain containing 4
SKP2	S-phase kinase-associated protein 2
EZH2	Enhancer of zeste homolog 2
CDH1	Cadherin 1
E-cadherin	Epithelial cadherin
N-cadherin	Neural cadherin
H19	H19 imprinted maternally expressed transcript
MMP14	Matrix metalloproteinase 14
MMP-2	Matrix metalloproteinase-2
MMP-9	Matrix metalloproteinase-9
H2AK5ac	Histone H2A lysine 5 acetylation
H3K4ac	Histone H3 lysine 4 acetylation
H3K9ac	Histone H3 lysine 9 acetylation
H3K23ac	Histone H3 lysine 23 acetylation
H3K27ac	Histone H3 lysine 27 acetylation
H4K16ac	Histone H4 lysine 16 acetylation
acetyl-CoA	Acetyl coenzyme A
SFN	Sulforaphane
ECN	Erucin
EGCG	Epigallocatechin-3-gallate
FK228	Romidepsin
TSA	Trichostatin A
TSC	Trichostatin C
tNOX	Tumor-associated NADH oxidase
CETSA	Cellular thermal shift assay
MCP30	Momordica charantia protein 30
LNCaP	Lymph node carcinoma of the prostate
MYST	MOZ, Ybf2/Sas3, Sas2, Tip60
